# Significant Improvement in Cognition in Mild to Moderately Severe Dementia Cases Treated with Transcranial Plus Intranasal Photobiomodulation: Case Series Report

**DOI:** 10.1089/pho.2016.4227

**Published:** 2017-08-01

**Authors:** Anita E. Saltmarche, Margaret A. Naeser, Kai Fai Ho, Michael R Hamblin, Lew Lim

**Affiliations:** ^1^ Saltmarche Health & Associates, Inc., Orangeville, Ontario, Canada.; ^2^ VA Boston Healthcare System, Boston, Massachusetts.; ^3^ Department of Neurology, Boston University School of Medicine, Boston, Massachusetts.; ^4^ STAT-TU, Inc., Toronto, Ontario, Canada.; ^5^ Harvard Medical School, Boston, Massachusetts.; ^6^ Wellman Center for Photomedicine, Massachusetts General Hospital, Boston, Massachusetts.; ^7^ Vielight, Inc., Toronto, Ontario, Canada.

**Keywords:** dementia, Alzheimer's disease, photobiomodulation, LLLT, LED, transcranial, intranasal

## Abstract

***Objective:*** This study investigated whether patients with mild to moderately severe dementia or possible Alzheimer's disease (AD) with Mini-Mental State Exam (MMSE) Baseline scores of 10–24 would improve when treated with near-infrared photobiomodulation (PBM) therapy. ***Background:*** Animal studies have presented the potential of PBM for AD. Dysregulation of the brain's default mode network (DMN) has been associated with AD, presenting the DMN as an identifiable target for PBM. ***Materials and methods:*** The study used 810 nm, 10 Hz pulsed, light-emitting diode devices combining transcranial plus intranasal PBM to treat the cortical nodes of the DMN (bilateral mesial prefrontal cortex, precuneus/posterior cingulate cortex, angular gyrus, and hippocampus). Five patients with mild to moderately severe cognitive impairment were entered into 12 weeks of active treatment as well as a follow-up no-treatment, 4-week period. Patients were assessed with the MMSE and Alzheimer's Disease Assessment Scale (ADAS-cog) tests. The protocol involved weekly, in-clinic use of a transcranial-intranasal PBM device; and daily at-home use of an intranasal-only device. ***Results:*** There was significant improvement after 12 weeks of PBM (MMSE, *p* < 0.003; ADAS-cog, *p* < 0.023). Increased function, better sleep, fewer angry outbursts, less anxiety, and wandering were reported post-PBM. There were no negative side effects. Precipitous declines were observed during the follow-up no-treatment, 4-week period. This is the first completed PBM case series to report significant, cognitive improvement in mild to moderately severe dementia and possible AD cases. ***Conclusions:*** Results suggest that larger, controlled studies are warranted. PBM shows potential for home treatment of patients with dementia and AD.

## Introduction

Declining memory, cognition, and quality of life (QoL) are symptoms associated with most forms of dementia and Alzheimer's disease (AD). An estimated 5.4 million Americans are living with AD, adding a new case every 66 sec. In addition, 15.9 million families and friends provide 18.1 billion hours of unpaid care, with an estimated $221.3 billion economic value. In 2016, AD and other dementias will cost the US $236 billion. The annual number of new cases of Alzheimer's and other dementias is projected to double by 2050.^1^ In 2015, an estimated 46.8 million people worldwide lived with dementia, with a projected cost of $1 trillion by 2018.^2^ The failure of numerous clinical trials with new pharmaceuticals^3^ underlines the need for newer, safer alternative treatments. Therefore, an effective treatment for dementia and AD would have enormous socioeconomic impact.

The relatively small number of new pharmaceutical compounds entering clinical trials to treat AD suggests that there is insufficient drug discovery activity. A number of those in advanced trials have been repurposed, which reduces the expectation of a novel pharmaceutical introduction to alter the status quo.^3^ New AD research and development are trending toward treating early disease stages even before the onset of dementia symptoms.^4^ Although there are still drug developments to treat all stages of AD, the pipeline for those treating the disease with dementia onset at advanced stages is fast shrinking.^3^ For example, the recently reported success with aducanumab to reduce brain amyloid beta (Aβ) in patients could lead to a drug targeting only early stage AD.^5^ This trend leaves little to address “mild to moderately severe” AD, which constitutes a large portion of the AD patient population. Any prospect of a viable treatment for more advanced-stage AD would be important.

Photobiomodulation (PBM) therapy is a safe, non-invasive, and non-thermal modality that is based on a strong body of research dating back to the 1960s. Also known as low-level laser (or light) therapy, it uses either visible red or near-infrared (NIR) light to stimulate, heal, and repair damaged or dying tissue cells. The mechanisms of action involve the stimulation of mitochondria by the absorption of photons in cytochrome c oxidase, resulting in increased adenosine triphosphate production, reduced oxidative stress, anti-inflammatory effects,^6^ and increased focal cerebral blood flow.^7,8^ In addition to a local effect observed when light of selected parameters are directed to the injured or damaged area, there is also a systemic effect where wounds located distally from the point of application also show improved wound healing.^9^ A study showed that delivering PBM to the tibia (stimulating bone marrow and mesenchymal stem cells) was associated with a 35% increase in phagocytosis of Aβ and a significant reduction in Aβ brain burden, promoting beneficial behavioral effects in a mouse model of AD.^10^ Along these lines, one could expect PBM to be delivered to tissues and the cranium surrounding the brain to have a similar effect, but with more efficient transcranial delivery to the brain due to the closer proximity of the PBM device to the skull. Several animal studies using transcranial PBM have shown positive outcomes in mouse models with neurodegenerative diseases such as AD.^11–14^ and Parkinson's disease.^15–17^ These results encourage using transcranial PBM to treat patients diagnosed with dementia or AD.

The pathology of AD originates in the lateral entorhinal cortex of the hippocampus, and it later progresses to widespread areas of association cortex.^18^ The widely recognized underlying neuropathology in AD is the deposition of Aβ and the accumulation of hyperphosphorylated tau protein.^19^ With progression, there is also functional dysregulation of the intrinsic cortical network, the default mode network (DMN).^20–23^ The cortical nodes of the DMN include bilateral hippocampus (entorhinal cortex), mesial prefrontal cortex (mPFC), precuneus/posterior cingulate cortex (precun/pCC), and inferior parietal lobe (angular gyrus).^24^ The temporally coordinated balance between deactivation/activation of the DMN and other intrinsic cortical networks is highly dysregulated in AD, and this worsens over time as the disease progresses. Hence, treatment of cortical nodes in the DMN would be a reasonable treatment goal. Treatment of a limited number of cortical target areas lends itself to engineering/development of PBM devices that have two advantages: First, they are efficient (delivering an adequate dose of photons without increasing heat to a specific cortical area) and second, they are safe and practical, being suitable for future home treatment use.

This is a case series report of five patients treated over a 12-week period with PBM, presenting its effect on patients with “mild to moderately-severe” dementia, or AD. The devices used were wearable, transcranial, and intranasal home-use PBM devices, using pulsed, 810 nm light-emitting diodes (LEDs) that emit non-coherent light (vs. lasers that emit coherent light).

## Materials and Methods

### Patients

The patients were recruited through advertisements placed in newspapers distributed in Toronto and Orangeville, Ontario, Canada, enlisting people with a diagnosis of dementia or AD. In this article, they are labeled as Patients 1–5, in order of degree of severity on the Mini-Mental State Exam (MMSE) as tested at baseline (Table 1). All patients had been diagnosed with dementia or AD by their physicians. Their ages were 72–90 years [mean 77.6; standard deviation (SD) 7.23]; the time between their diagnoses and participation in the study ranged from 6 months to 8 years (mean 3.2 years; SD 2.88).

**Table 1.** Demographics and Baseline Characteristics of Each Patient

*Patient no.*^a^	*Baseline MMSE score*^b^	*Baseline ADAS-cog score*	*Age at entry*	*Gender*	*Dementia diagnosis (years)*	*Diagnosis from physician*	*Years of education*	*Prescribed dementia medication*
1	10	58	77	Female	2	Dementia	7	No
2	10	58	90	Male	2	Dementia	10+ apprentice	Donepezil
3	21	26.33	76	Male	0.5	Dementia. Memory changes noted by wife 1 year earlier.	16	No
4	22	20.67	72	Male	3.5	Dementia. Very gradual decline, works part-time.	10	Donepezil
5	24	14.33	73	Male	8	Dementia. Diagnosis by one physician, AD. Failed re-registration exam.	18	Donepezil
Mean (SD)	17.4 (6.84)	35.47 (21.00)	77.6 (7.23)		3.2 (2.89)		12.2 (4.6)	

^a^ The patients are ranked by severity of impairment represented by MMSE scores.

^b^ The Alzheimer's Association categorizes MMSE scores as follows: severe, <12; moderate, 13–19; and mild, 20–24.^26^

ADAS-cog, Alzheimer's disease assessment scale-cognitive subscale; MMSE, Mini-Mental State Exam; SD, standard deviation.

Before enrollment into the study, informed consent was obtained from the participants and signed in the presence of accompanying caregivers. The protocol and Informed Consent Forms (ICF) were reviewed by Health Canada. The subjects were not paid for participation in the study. However, the ICF included a statement that patients who completed the study would be given their own PBM devices to keep for home use.

### Methods

In this study, five patients with “mild to moderately-severe” dementia or AD were recruited to be treated with near infrared (NIR) PBM at 810 nm wavelength pulsed at 10 Hz, during a “12-week, Active-Treatment Period.” The effect of withdrawal of PBM treatment was observed in a subsequent “4-week, No-Treatment Period” ending after week 16, which also concluded the study. Throughout the treatment period, the safety of the PBM treatment was evaluated with weekly in-clinic visits and a daily home treatment journal.

### Cognitive outcome measures

Patients entered the study after having been previously diagnosed with dementia or AD, and scores of 10–24 on the MMSE.^25^ MMSE scores in the range of 10–24 are considered “mild to moderately-severe” dementia in this study. This definition broadly follows guidance from the Alzheimer's Association^26^ and had been adopted in several pharmaceutical clinical studies, which associated this range of MMSE scores with “mild to moderate Alzheimer's disease.”^27–30^

The MMSE, and the cognitive behavior scale from the Alzheimer's Disease Assessment Scale (ADAS-cog) were used to measure any changes in cognition during the treatment series. These scales are widely administered in AD treatment trials. The MMSE is scored on a scale of 0–30, where higher scores are indicative of better cognitive function. The ADAS-cog is scored between 0 and 70, where higher scores are indicative of more cognitive impairment. Patients were tested three times during the “Active Treatment 12-week period,” starting at baseline (week 0), mid-treatment point (week 6), and at the end of treatment (week 12). The test intervals at week 6 and week 12 were selected based on the precedence of a pivotal pharmaceutical (donepezil) study.^30^ This was followed by a final “4-Week, No-Treatment Period” when all PBM devices were withdrawn, from the end of week 12 through the end of week 16. Qualitative feedback from the patients or family caregivers was documented during in-clinic interviews and from a “Daily Home Treatment Journal.”

### PBM devices and treatment protocol

**FIG. 1.**
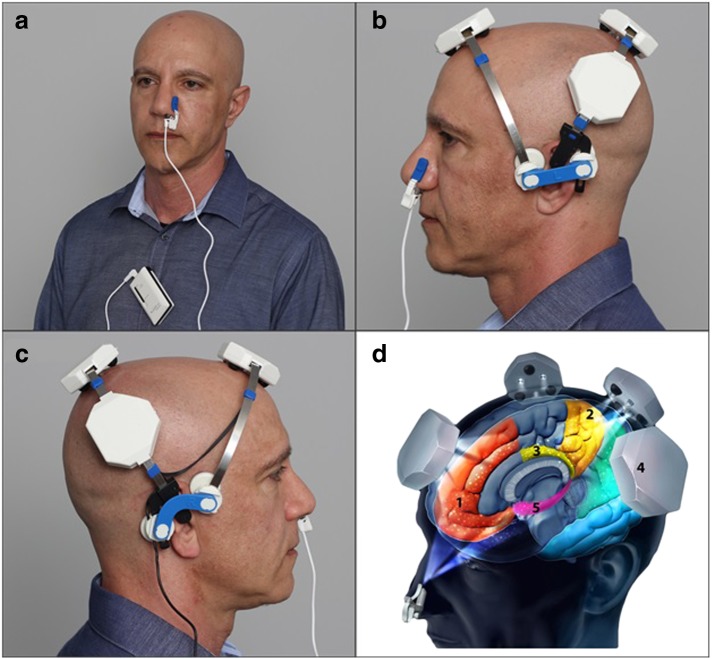
Photographs of Vielight “810” and “Neuro” illustrating correct device positions for treatment, and corresponding targeted network hubs. **(a)** Vielight “810.” **(b)** Vielight “Neuro,” left view. **(c)** Vielight “Neuro,” right view. **(d)** Targeted default mode network nodes: (1) Mesial prefrontal cortex, (2) Precuneus, (3) Posterior cingulate cortex, (4) Inferior parietal lobe, and (5) Hippocampus.

Two types of painless, non-invasive, non-thermal, non-laser, LED PBM devices were used during the study: the intranasal-only “810” device (Fig. 1a) and the “Neuro” device (Fig. 1b–d) provided by Vielight, Inc. (Toronto, Canada). The devices were not labeled for treating dementia or AD and would be described as non-regulated, “low risk general wellness products,” according to the Food and Drug Administration document, “General Wellness: Policy for Low Risk Devices,” released on July 29, 2016.

The separate intranasal-only “810” device (Fig. 1a), used only at home, consisted of one diode, which emitted NIR light of 810 nm wavelength, pulsed at 10 Hz, 50% duty cycle. It shut off automatically after 25 min of treatment time, operating on a single AA battery. See Table 2 for specifications and parameters.

**Table 2.** Vielight Intranasal-Only “810” and “Neuro” Parameters

	*“810” Intranasal device*	*“Neuro” transcranial-intranasal device*
Source	LED	LED
Wavelength, nm	810	810
Power output, mW	14.2	41 (transcranial) 23 (intranasal)
Power density per LED, mW/cm^2^	14.2	41 (transcranial) 23 (intranasal)
Pulse frequency, Hz	10	10
Pulse duty cycle, percentage	50	50
Duration of each treatment session, minutes	25	20
Beam spot size, cm^2^	≈1	≈1
Energy delivered, Joules	10.65	24.6 (transcranial) 13.8 (intranasal)
Energy density per LED, J/cm^2^	10.65	24.6 (transcranial) 13.8 (intranasal)
Cumulative energy density per LED, per week during weeks 1 and 2, J/cm^2^	53.25	49.2 (transcranial) 27.6 (intranasal)
Cumulative energy density per LED, per week during weeks 3 to 12, J/cm^2^	63.90	24.6 (transcranial) 13.8 (intranasal)
Dose of each treatment session, Joules	10.65	309
Cumulative dose per week during weeks 1 and 2, Joules	639 total	
Cumulative dose per week during weeks 3 to 12, Joules	375 total	

The 14.2 mW/cm^2^ power density for the “810” intranasal device is similar to the one used in previous research (650 nm wavelength, 8.32 mW/cm^2^, used daily for 30 min for 20 days, 10 days on, 3 days off, then 10 days on). That research demonstrated efficacy for improving blood lipid levels and rheology of the blood; there were no negative side effects.^32^ Based on that research and our clinical experience with the intranasal device, the daily intranasal treatments were deemed to be safe. The “Neuro” delivers 41 mW/cm^2^, which is much less than the 250 mW/cm^2^ used in research by Schiffer et al.^7^ but almost twice the transcranial power density used by Naeser et al. (22 mW/cm^2^).^33^ Both research studies demonstrated efficacy, and no negative side effects were present.^33^ There is no method to measure/calculate the loss of energy in the transmission of light through living tissues. It is a biological fact, however, that the scalp and hair are major barriers. To compensate for this, the transcranial diodes in the “Neuro” had almost twice the power density (41 mW/cm^2^) than the single intranasal diode (23 mW/cm^2^). A recent transcranial study with human cadaver brains has measured the penetration of near-infrared photons (808 nm) to a depth of 40 mm.^34^

LED, light emitting diode.

The “Neuro” consisted of a headset frame, holding four separate LED cluster heads plus one intranasal LED. All diodes emitted light of 810 nm wavelength, synchronized to pulse at 10 Hz, 50% duty cycle (Fig. 1b and c). The device shut off automatically after 20 min of treatment time (powered by rechargeable NiMH batteries). Each of the four LED cluster heads on the headset contained three LEDs. The “Neuro” also consisted of a single intranasal diode (Fig. 1b) with higher power than the intranasal-only “810” device. See Table 2 for the “Neuro's” specifications and parameters.

The “Neuro” was used only during the in-clinic site visits during the “12-week, Active Treatment Period.” It was applied 2 × per week during in-clinic visits for the first 2 weeks, and then applied only 1 × per week, for each of the next 10 weeks. Participants were treated in a sitting or reclining position with the LED clusters securely positioned on the head.

### Home treatment protocol

The intranasal-only “810” was used at home, daily during the “12-week, Active-Treatment Period,” except on a day when the participant visited the clinic for treatment with the “Neuro” device. To encourage adherence to the home-treatment PBM regimen, each treatment was recorded in the “Daily Home Treatment Journal.” In addition, changes in memory, cognition, QoL, or general health conditions were noted in the comments section of the journal and reviewed at each in-clinic appointment. The participants were also monitored for clinical safety and any adverse events.

## Results

**FIG. 2.**
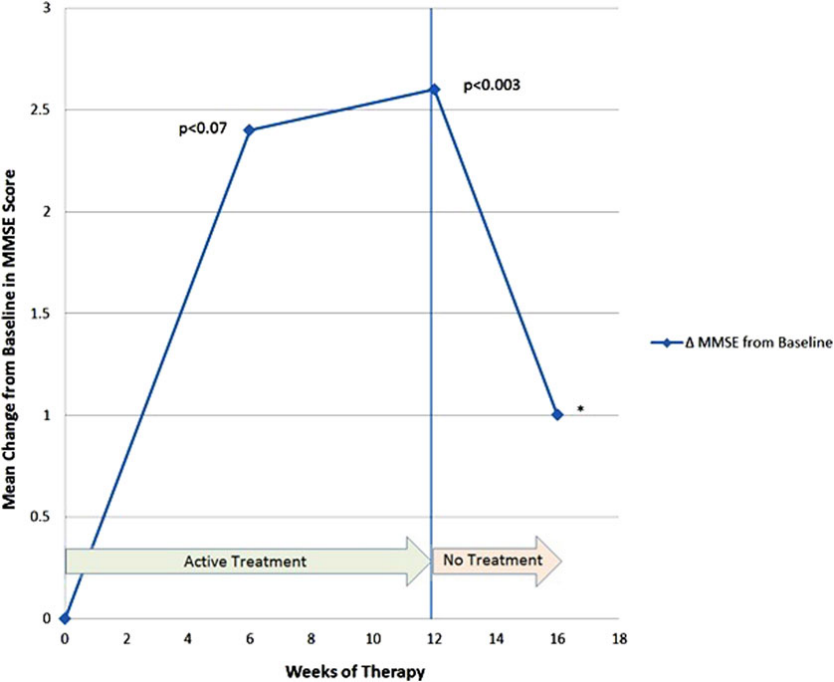
Mean change from baseline in MMSE scores. Higher numbers indicate better cognition on this test. *The *p* value for week 16 is omitted due to missing data from a patient who dropped out during the “4-Week, No-Treatment Period.” MMSE, Mini-Mental State Exam.

**FIG. 3.**
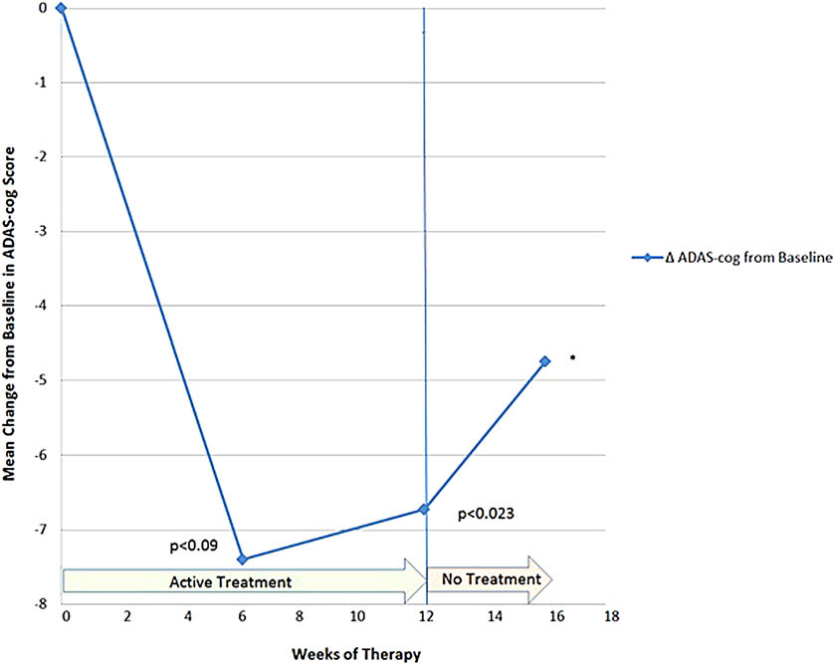
Mean change from baseline in ADAS-cog scores. Lower numbers indicate better cognition on this test. *The *p* value for week 16 is omitted due to missing data from a patient who dropped out during the “4-Week, No-Treatment Period.” ADAS-cog, Alzheimer's disease assessment scale-cognitive subscale.

The results are shown in Table 3 and Figs. 2 and 3. After 12 weeks of PBM treatments, there were significant improvements on the MMSE (mean +2.60 points, *p* < 0.003, two tailed) and the ADAS-cog (mean −6.73 points, *p* < 0.023, two tailed). At baseline, the mean (SD) for MMSE and ADAS-cog scores were 17.4 (6.84) and 35.47 (21.00), respectively. At the end of week 12, the mean scores improved to 20.00 (7.10) on the MMSE, and to 28.73 (18.85) on the ADAS-cog.

**Table 3.** Results of Mini-Mental State Exam and Alzheimer's Disease Assessment Scale-Cognitive Subscale Scores for Each Case and Mean Changes from Baseline

	*Baseline*		*Week 6*		*Week 12*		*4-Week, no treatment*	
*Patient no.*	*MMSE*	*ADAS-cog*	*MMSE*	*ADAS-cog*	*MMSE*	*ADAS-cog*	*MMSE*	*ADAS-cog*
1	10	58	11	52	13	50	11	52
2	10	58	13	46	12	48.67	Dropped out^a^	Dropped out^a^
3	21	26.33	27	9.33	23	16.66	20	22
4	22	20.67	23	15.66	24	13.33	24	14
5	24	14.33	25	17.34	28	15	25	12.33
Mean (SD)	17.40 (6.84)	35.47 (21.00)	19.80 (7.29)	28.07 (19.46)	20.00 (7.11)	28.73 (18.85)	20.25 (6.60)^b^	25.08 (18.44)^b^
Mean change from baseline	0	0	2.40	−7.40	2.60	−6.73	1.00^b^	−4.75^b^
*p* Value of mean change			<0.07	<0.09	<0.003	<0.023	^c^	^c^

The changes in the mean scores from baseline are also shown in Figures 2 (MMSE) and 3 (ADAS-cog).

^a^ Patient 2 experienced rapid cognitive decline soon after entering the 4-Week No-Treatment Period. We acceded to the family's request to disrupt this control and allowed him back on treatment. He was not scored for this period.

^b^ The Mean for the 4-Week No-Treatment Period is calculated based on incomplete data, without Patient 2.

^c^ The *p* value was omitted for the 4-Week No-Treatment Period due to incomplete data, without Patient 2.

During the “4-Week, No-Treatment Period,” Patient 1 complained about the return of previous problems, but remained in the study until week 16, when testing could be completed. Her data at week 16 showed a decline in both the MMSE and the ADAS-cog scores. Patient 2 dropped out of the study after only 1 week into the “4-Week, No-Treatment Period,” when “precipitous cognitive and functional decline” were reported by the family. Because this was causing a high level of emotional distress for family and patient, the authors made the decision to disrupt his participation in the study. The family was then given an active, intranasal-only “810” device and the “Neuro” device for home use, despite not completing the study. Later, the family reported anecdotally that behavioral improvements resumed. There are no cognitive test data for Patient 2, at week 16.

After the “4-Week, No-Treatment Period,” compared with their scores after 12 weeks of PBM therapy, the MMSE scores were worse by 2 or 3 points, for three of the four cases; and about the same, for the fourth case. Likewise, for the ADAS-cog scores after the “4-Week, No-Treatment Period,” relative to their scores after 12 weeks of PBM therapy, the ADAS-cog scores were worse by 2 and 5 points for two cases, about the same for one case, and better by 3 points for one case. Thus, only one patient continued to improve on one test (ADAS-cog) after the “4-Week, No Treatment period” (Patient 5), but he worsened on the MMSE at that time.

Collectively, the patients improved at the fastest rate during the first 6 weeks of PBM therapy, with a trend to significant improvement at that time for both the MMSE (*p* < 0.07, two-tailed), and for the ADAS-cog (*p* < 0.09, two-tailed) (Table 3). The significant *p* values of <0.003 (MMSE) and <0.023 (ADAS-cog) were observed after 12 weeks of active PBM treatments. The improved test scores were supported by improved QoL feedback from patients and caregivers (Table 4). Improvements were noted in the areas of functional abilities (i.e., decreased incontinence, increased mobility), sleep, fewer angry outbursts, less anxiety, and wandering. Caregivers expressed a better QoL for themselves during the active treatment period when behavior was improved in the patients.

**Table 4.** Quality of Life and Functional Changes from Baseline, During 12-Week Treatment Period, and After 4-Week No-Treatment Follow-Up Period, Reported By Participant and Families

*Patient no.*	*Baseline*	*12-week treatment period*	*No-treatment follow-up period*
1	Apprehensive, spoke predominantly Portuguese with family, complained “her head felt too heavy to hold up, headache.” Only responded to questions. Family stated she was more anxious, had decreased ability to cook or clean, less interactive with family.	Openly smiling, laughing, hugged assessor. Stated frequently, head feels “lighter” “clearer,” no headache. Family stated, “more talkative and active” (i.e., cooking, cleaning, going for walks, answering phone). Able to give a recipe to assessor by memory.	Progressively more withdrawn, less engaged. More tired, feeling “cloudy” “heavy head,” headaches returned. Cooked and cleaned less, personal hygiene declined. Did not want to participate in family gatherings.
2	Infrequent eye contact with assessor. Predominantly answered in Italian (native language) with long pauses between questions. Stooped posture, shuffling gait, live-in caregiver, assisted with mobility, dressing, personal hygiene, incontinent 6/7 nights. Not initiating conversation, minimal engagement during family visits. Did not discuss his wife's death.	Looked directly at assessor, spoke predom-inantly English, humorous, and smiling. Remembered assessor's name, reason for visit and stated, “doing better.” By week 6, walked into office more upright, at steady pace, independently transferred from chairs. Incontinent 1–2/7 nights. Occasionally dressed independently, more communicative, happier with caregiver and family. Acknowledged wife's death and able to speak to family.	First week without PBM treatment, rapidly declined in behavior (uncooperative and belligerent); functional decline (required assistance with mobility, hygiene, and dressing); and cognitive decline (less able to follow conversation, respond appropriately, or remember events). Family requested to have LED treatment resumed.
3	Humor was used to compensate for inability to answer questions. Denied memory loss. Thought he was still working. Read and listened to news. Wife not sure what he remembered. Minimal discussion of news or events.	Patient stated, “easier to answer test questions,” recognized when unable. Wife stated he was more interactive and was reading his professional publications. Week 10, foot ulcer returned, below-the-knee edema, erythema, pain, grimaced with transfers from chair, and less bright and interactive.	Patient treated at foot clinic, little change. Had foot pain all of the time, leg edema below the knee. Less focused during testing, decreased interaction, less humorous, and personal hygiene declined (e.g., not clean shaven).
4	Used to be outgoing, humorous, but then felt less happy. Agreed when wife stated that he was becoming more forgetful (i.e., only drove on familiar routes and misplaced items). Asked wife for test answers. Working part-time, cooks his own ethnic meals.	Returned to building “found object sculptures.” Able to re-route driving to accommodate traffic, becoming less forgetful, needed fewer reminders. Less dependent on wife for “entertainment,” generally happier. Looked less to the wife for test answers, laughed, then answered independently.	No decline during “No Treatment” period. Wife confirmed husband had not lost the gains achieved during treatment.
5	Patient open about loss of memory and diagnosis of AD. Interactive, but slightly reserved. Aware when unable to answer test questions, needed prompting to provide answer. Stated he and his wife continue to live a full life, but the future was scary.	Week 3, stated he felt brighter, world had more color, forgot less frequently as to why he went into a room. Worked in garden with wife, preparing to start oil painting again. More humorous, interactive, less hesitant during testing. Wife (nurse) stated she was pleased with positive changes.	Gradual decrease in “brightness and clarity.” Both patient and wife noticed decline in memory, focus, less able to initiate and complete tasks independently.

AD, Alzheimer's disease.

For all patients throughout the 12 weeks of active treatment, the PBM intervention was well tolerated, with no report of any adverse events. To our knowledge, this case series is the first completed PBM case series to document significant, cognitive improvement in mild to moderately severe dementia or AD.^31^

## Discussion

In the five mild to moderately severe dementia cases who participated in this transcranial plus intranasal PBM study, significant improvements were present in cognition after 12 weeks of active treatment (+2.60 points on the MMSE, *p* < 0.003; and −6.73 points on the ADAS-cog, *p* < 0.023). After 6 weeks of PBM therapy, there was a trend toward significant improvement, with +2.40 points on the MMSE (*p* < 0.07), and with −7.40 points on the ADAS-cog (*p* < 0.09). The change in ADAS-cog scores of −7.40 after 6 weeks of PBM therapy compares favorably with the change in ADAS-cog scores from a large dementia study where the pharmaceutical donepezil was used to treat mild-to-moderate AD patients. In that study, a dose of 10 mg/day produced a mean cognitive change of −1.06 points on the ADAS-cog from baseline to week 6.^30^ Further, comparisons to the final end-point of the donepezil study were not possible, however, because patients in the present PBM study were not treated out to 24 weeks. There were no reported side effects in the present PBM study such as diarrhea, nausea, vomiting, anorexia, or dizziness.

In the present PBM study, after the “4-Week, No-Treatment Period,” 3 out of 4 of the participants worsened on their MMSE scores, relative to their scores after the “12-Week, Active Treatment Period”; and 2 out of 4 of them worsened on the ADAS-cog scores, relative to scores after the “12-Week, Active Treatment Period.” Only one patient continued to improve on one test (ADAS-cog) after the “4-Week, No-Treatment Period” (Patient 5), but he worsened on the MMSE at that time. Deterioration in the MMSE and ADAS-cog scores after the “4-Week, No-Treatment Period” is supportive of the significant improvements present after the 12 weeks of active PBM therapy. Deterioration in function and behavior was so marked in one patient (Patient 2) after only 1 week of no treatment that the authors returned the PBM equipment to the family at that time, without further follow-up testing. The family later reported anecdotally that improvements resumed.

In addition to significant improvements in cognition after 12 weeks of active treatment, the families and patients reported better QoL. Improvements were noted in functional abilities (i.e., decreased incontinence and increased mobility): the areas of sleep, fewer angry outbursts, less anxiety, and wandering.

In this study, NIR light was targeted to specific cortical nodes of the DMN using only a few LEDs. The DMN areas included bilateral mPFC, precun/pCC, angular gyrus, and hippocampus—areas associated with pathology in AD,^18,19^ and with DMN dysregulation in AD^20–23^ (Fig. 1d). It is likely that the NIR photons dispersed somewhat, after specific scalp application; however, it is hypothesized that relatively more photons reached the targeted cortical nodes than other non-targeted cortical areas. Significant cognitive improvements after the active, transcranial plus intranasal PBM therapy support the notion that functional connectivity was likely strengthened among the nodes within the DMN post-PBM. Resting-state functional-connectivity magnetic resonance imaging scans would be necessary, however, pre- and post-PBM to support this notion.

The improvements observed in this study support expectations from past animal studies with AD models.^11–14^ Although we could not feasibly observe changes in the AD biomarkers in human subjects in the same way, we could report changes as presented in the cognitive scale test scores.

### Considerations for future studies

1. The “4-Week, No-Treatment Period” resulted in an erosion of the positive effects of the PBM therapy achieved during the 12 weeks of active treatment. This was unexpected and difficult for both the participants and the caregivers. A future study should consider avoiding discontinuation of active PBM once it has been started, especially in progressive, neurodegenerative diseases such as AD.

2. Traveling to the clinic sites was stressful. Adherence to the home treatment protocol was high, as evidenced with the “Daily Home Treatment Journal.” Participants and caregivers stated that the intranasal-only “810” device was easy to use at home. The “Neuro” could also be easily used at home, providing caregivers and patients control of their own treatments. Future studies may consider home PBM therapy, combined with telemetry and video conferencing to monitor treatments (after initial in-person training), and in-person clinic visits would only be necessary for cognitive assessments.

3. Standardized cognitive assessments (MMSE and ADAS-cog) were used in this study. Future research should consider adding other quantitative, standardized methods for documenting changes—that is, improved sleep, communication, and social interaction; decreased anxiety, depression, and disruptive behaviors (angry outbursts, physical aggression, or wandering).

### Limitation of this study

The main limitation of this study is the small number of patients, and there was no comparable placebo-treated group. Although the outcomes in this group of dementia patients are encouraging, future studies will be important.

## Conclusions and Summary

Results from this small study suggest that transcranial plus intranasal NIR PBM therapy may be safely used with mild to moderately severe dementia and AD (baseline MMSE of 10–24). Results showed significant improvement in cognition, functional abilities for daily living, and improved QoL. PBM was very well tolerated, exhibiting no adverse effects. The treatments likely need to be continued, however, on a regular, long-term basis. This suggests the importance of having PBM devices that are amenable to home use for treating dementia and AD. Results suggest that large-scale, controlled studies with homogeneous populations are warranted.
